# Development of Anti-CEA C_H_2 Domain-Deleted Antibody (M5A∆C_H_2) for the PET Imaging of Colorectal Cancer

**DOI:** 10.1007/s11307-025-01997-3

**Published:** 2025-03-14

**Authors:** Jitender Jitender, Teresa Hong, Anakim Sherman, Patty Wong, Eric Aniogo, Maciej Kujawski, John E. Shively, Paul J. Yazaki

**Affiliations:** Department of Immunology and Theranostics, Beckman Research Institute, City of Hope, USA

**Keywords:** ImmunoPET imaging, Anti-CEA antibody, Colorectal cancer, Antibody engineering, Radiodiagnostics

## Abstract

**Purpose:**

Recombinant antibody fragments represent a novel class of in vivo biological immunoPET imaging agents. This study developed a series of anti-carcinoembryonic antigen (CEA) C_H_2 domain-deleted antibodies to evaluate their rapid, high-level tumor targeting combined with fast blood clearance for immunoPET imaging in two colorectal cancer mouse models.

**Procedure:**

A series of humanized anti-CEA M5A∆C_H_2 recombinant antibody fragments were synthesized via transient mammalian expression and purified using a two-step process. The M5A∆CH2 antibody series was characterized by HPLC-SEC, SDS-PAGE and binding affinities. The M5A∆C_H_2-C5 antibody, which has five disulfide bridges in the modified IgG1/IgG3 hinge domain, was selected for positron emission tomography (PET) imaging. Site-specific thiol conjugation of the reduced hinge disulfides with the 1,4,7,10 tetraazacyclododecane-1,4,7-triacetic acid trisodium salt-vinyl sulfone (DO3A-VS) chelate was performed, followed by labeling with [^64^Cu-CuCl_2_]. The [^64^Cu]Cu-DO3A-M5A∆C_H_2-C5 was evaluated for CEA-positive tumor PET imaging at serial time points, pharmacokinetics and a terminal biodistribution study conducted in two CEA-positive colorectal cancer mouse models.

**Results:**

The anti-CEA M5A∆C_H_2 antibodies had high expression, were purified using a new CH3 domain affinity resin and were stable up to one year. ImmunoPET imaging and biodistribution studies were performed in athymic mice bearing human colorectal cancer LS174T tumors and immunocompetent transgenic-CEA (Tg-CEA) mice bearing MC-38 tumors transfected with the human CEA gene. The [^64^Cu]Cu-DO3A-M5A∆C_H_2-C5 showed rapid, high tumor localization and the expected fast blood clearance. Conclusions: A series of humanized anti-CEA M5A∆C_H_2 antibodies were designed for immunoPET imaging of colorectal cancer, and the [^64^Cu]Cu-DO3A-M5A∆C_H_2-C5 showed high tumor targeting and fast blood clearance supporting its potential for clinical trials.

**Supplementary Information:**

The online version contains supplementary material available at 10.1007/s11307-025-01997-3.

## Introduction

Molecular imaging has emerged as a non-invasive and quantitative technique for detecting cancer, monitoring its progression, and evaluating therapeutic responses by specific molecular targeting. ImmunoPET imaging leverages the unique properties of antibodies to selectively target antigens on cancer cells, providing quantitative data to support tumor localization, extent of disease, tumor heterogeneity, and therapeutic response. These detailed insights gained from immunoPET imaging aid in the accurate diagnosis and effective oncology treatment management. One such tool is the radiolabeled humanized hT84.66-M5A (M5A) monoclonal antibody (mAb) with high specificity and affinity toward carcinoembryonic antigen (CEA), a well-characterized tumor-associated antigen [33]. CEA is prominently overexpressed in various gastrointestinal (GI) cancer, including colorectal cancer, making it an ideal target for both diagnostic and therapeutic interventions [12, 25]. The radiolabeled M5A mAb is currently being evaluated in the clinic as a [^64^Cu]PET imaging agent [29] and for [^90^Y]radioimmunotherapy [1]. The continued development and refinement of M5A-based imaging and therapeutic strategies hold promise for enhancing the precision and efficacy of cancer management.

The utility of tumor targeting can be further augmented through antibody engineering approaches to optimize affinity and pharmacokinetic properties. Recombinant antibody fragments, such as single-domain antibody (sdAb, VHH or nanobody), single-chain fragment variable domain (scFv), Fab, F(ab’)_2_, diabody, minibody, and scFv-Fc have emerged as powerful tools in oncology imaging [2, 27]. These fragments, derived from conventional monoclonal antibodies, retain high specificity and affinity for their target antigens while offering advantages over full-length antibodies such as enhanced tumor penetration and accelerated clearance from non-target tissues [21]. The rapid pharmacokinetics of engineered antibody fragments allow for earlier imaging and superior contrast [30]. In addition, the engineering flexibility of these fragments also facilitates their conjugation with various imaging agents, such as radionuclides and fluorescent dyes. However, these benefits come with certain trade-offs. Small antibody fragments typically have shorter circulation times, which may limit their accumulation in tumors and reduce overall signal intensity. They also may lack the effector functions present in full-length antibodies, such as antibody-dependent cellular cytotoxicity (ADCC) and complement-dependent cytotoxicity (CDC), potentially diminishing their therapeutic efficacy.

Several anti-CEA T84.66 engineered antibody fragments have been evaluated in biodistribution studies targeting colorectal cancer in xenografted mice models. For instance, [^111^In] labeled anti-CEA T84.66 diabody demonstrated rapid clearance from blood and normal tissue while effectively targeting tumors [34]. Similarly, the cT84.66 minibody with an intermediate molecular weight (MW ~ 80 kDa) labeled with [^123^I] showed excellent tumor targeting and imaging properties in athymic mouse tumor xenograft model [28].

A critical aspect of enhancing the precision of imaging antibody conjugates lies in the method of conjugation. Non-specific conjugation procedures, which involve the stochastic ligation of amine-reactive prosthetic groups to surface lysines, produce heterogeneous conjugates with poor reproducibility and compromised binding capabilities [21]. In contrast, site-specific bioconjugation methods yield homogeneous conjugates with superior binding properties [22]. Site-specific conjugation of antibodies can be achieved via enzymatic (transglutaminase, glycan-mediated) or chemical (selective reduction of thiol bridges, N-terminal modification) methods. Among these, alkylation of cysteine is a widely used and conventional method for site-specific conjugation. The interchain cysteine residues in the hinge region can be selectively reduced using dithiothreitol (DTT) or Tris (2-carboxyethyl) phosphine (TCEP) under controlled conditions to achieve optimal payload to antibody ratio for enhanced potency [11, 19].

**Fig. 1:**
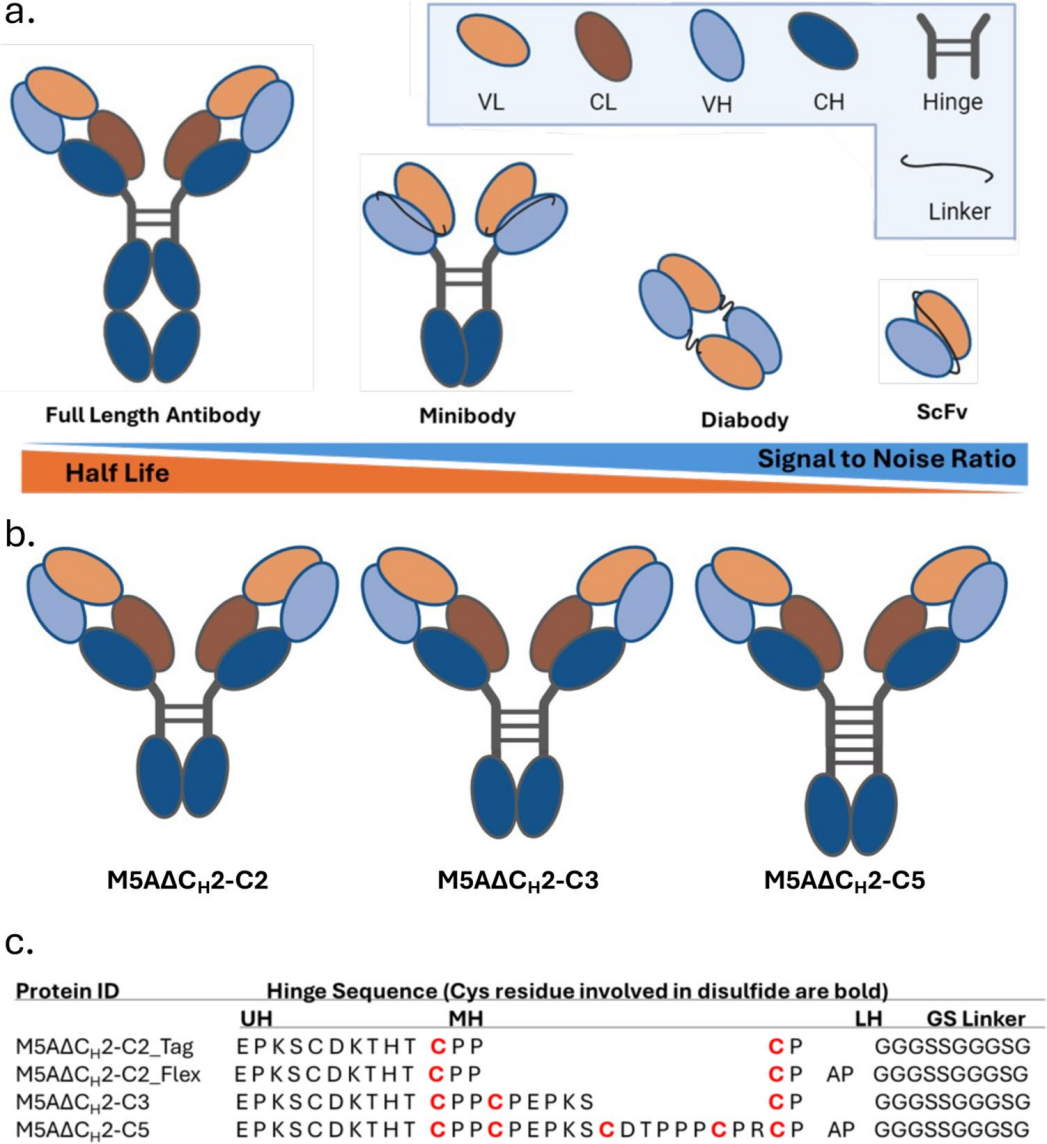
Design of anti-CEA M5AΔCH2 antibody constructs. **a** Full-length antibody and smaller recombinant antibody fragments developed to target CEA positive tumors [20, 28, 33]. Antibody fragments have demonstrated shorter half-life but better signal to noise ratio compared to full length antibody [30]. **b** M5AΔCH2 constructs were designed to include 2, 3 and 5 cysteine bridges in the hinge region. **c** Amino acid sequences of M5A∆CH2 hinge domain series. Cysteine involved in disulfide bond formation are represented in bold red. (CH: Constant heavy, CL: Constant light, LH: lower hinge, MH: middle hinge, UH: upper hinge, VH: Variable Heavy, VL: Variable light).

In this study we designed and expressed a series of C_H_2 domain-deleted antibodies (∆C_H_2) based on M5A. The M5A-∆C_H_2 antibodies feature modified hinge domains for conjugation of additional site-specific payloads and enhanced structural stability (Fig. 1). This work details the production and biochemical characterization of the anti-CEA ∆C_H_2 antibodies. Notably, the M5A∆C_H_2-C5 was labeled with [^64^Cu] (half-life 12.7 h., 0.653 MeV positron) and evaluated for PET imaging in two human colorectal cancer mouse models. The results showed rapid, high tumor uptake and fast blood clearance making it an excellent immunoPET imaging candidate for advancement toward clinical evaluation.

## Material and Methods

### Anti-CEA Delta C_H_2 Antibody Design and Production

The humanized anti-CEA hT84.66-M5A (M5A) mAb was engineered into a C_H_2 domain-deleted (∆C_H_2) mAb fragment format based on the anti-TAG-72 ∆C_H_2 antibody [23, 33]. The IgG1 heavy chain C_H_2 domain was replaced with a linker consisting of serine (S) and glycine (G) amino acids to join the IgG1 lower hinge to the C_H_3 domain. Two different linkers named Flex (APGGGSSGGGSG), and TAG (GGGSSGGGSG) were incorporated as previously described [10, 13]. Modifying the IgG hinge design of Glaser et al., four hinge and linker variants were developed having two (C2), three (C3) and five (C5) cysteine disulfide bridges (M5A∆C_H_2-C2_Tag, M5A∆C_H_2-C2_Flex, M5A∆C_H_2-C3_TAG, and M5A∆C_H_2-C5_Flex, respectively) as shown in Fig. 1 [10].

cDNA encoding the gene constructs were synthesized by GeneArt (ThermoFisher Scientific, MA). The individual pairs of light and heavy chain genes were subcloned into the pEE12/6 dual vector GS expression system (Lonza Biologics, Switzerland). Plasmids encoding the individual delta C_H_2 antibody constructs were transiently expressed using the ExpiFectamine™ 293 Transfection Kit (Gibco) as per manufacturer instructions. The cells were grown in Expi293 expression media at 125 rpm, 37 ˚C and 8% CO_2_ in a humidified incubator and harvested on day 6 post-transfection.

### Purification of M5A∆C_H_2 Antibodies

The cell harvests were clarified by centrifugation (3000 g for 10 min) and filtered to remove particulates. The cell-free harvest was treated with the strong anion AG1-X8 resin (Bio-Rad, Hercules, CA) (5% w/v) by incubation on a roller assembly at 4˚C overnight and sterile filtered to provide a clarified feed stream. Chromatography was performed using an NGC chromatography system (BioRad) controlled and recorded using ChromLab software (Bio-Rad). For affinity purification an AP-1 column (Waters, MA) was packed with 7 ml of Capture Select™ FcXP affinity matrix (ThermoFisher Scientific). The column was pre-equilibrated with 20 column volumes (CV) of phosphate buffered saline (PBS) pH 7.4 at 2 ml/min. The individual harvests were loaded onto the column at a flow rate of 1 ml/min. The column was washed with 5 CV PBS, 10 CV of a high salt buffer (0.02 M sodium phosphate, 0.02 M sodium citrate, 0.5 M NaCl, pH 7.5) and 5 CV of PBS. The M5A∆C_H_2 constructs were eluted with 0.1 M glycine, pH 3.0 buffer. Eluted protein was neutralized with 0.5 M [2-(*N*-morpholino)ethanesulfonic acid] (MES), pH 8.0 buffer to pH 6.5. The column was cleaned and sanitized with 1% phosphoric acid and 2 M guanidine hydrochloride and stored at 4˚C in 20% ethanol.

Ceramic Hydroxyapatite Type 1, 20 µm (CHT) (Bio-Rad) chromatography was used as polishing step to remove aggregates at a flow rate of 5 ml/min (except where stated otherwise). A 7 ml CHT column was pre-equilibrated with 10 CV of 0.05 M MES, 0.01 M potassium phosphate, pH 6.5 buffer. The purified antibody was loaded at 2.5 ml/min followed by 2 CV of wash buffer (0.05 M MES, 0.05 M potassium phosphate, pH 6.5). A 30 CV linear gradient of 100% wash buffer to 100% elution buffer (0.05 M MES, 0.2 M potassium phosphate, pH 6.5) was used to elute the protein. Monitoring elution by A_280_, peaks were collected and analyzed for the presence of aggregates by high performance liquid chromatography-size exclusion chromatography (HPLC-SEC) using a Superdex 200 10/300 column (Cytiva, Wilmington, DE). The peaks containing the monomeric form were combined, buffer exchanged with PBS using 10 kDa MWCO protein concentrators (ThermoFisher Scientific) and sterile filtered. M5A∆C_H_2 antibody fragments were stored at a concentration range of 6.5–7.5 mg/ml in PBS at 4˚C.

### Biochemical Analysis

Antibody samples were analyzed by SDS-PAGE under non-reducing and reducing conditions on 10% Mini Precast Protein Gels (Bio-Rad). Three micrograms of antibody samples were mixed with loading buffer with or without DTT reducing agent. The sample were heated for 5 min at 95˚C before loading and electrophoresed at 200 V × 30 min. The gels were imaged on a ChemDoc imaging system (Bio-Rad) and analyzed using the Image-Lab software (Bio-Rad). To compare the thiol stability of M5A∆C_H_2 constructs, the samples were incubated with TCEP for one hr at room temperature. TCEP: protein molar ratios of 30:1, 15:1, 7.5:1, and 3.5:1 were used. The samples were run on SDS-PAGE under non-reducing conditions. Stability was assessed at serial time points by HPLC-SEC analysis. Anti-CEA immunoreactivity was confirmed by incubating 10 µg of antibody with 50 µg of soluble CEA, (37˚C for 30 min) and analyzing for the formation of a 300 kilodaltons (kDa) antibody-antigen complex by HPLC-SEC [17].

Surface plasmon resonance (SPR) assays were performed on Biacore X100 (Cytiva) by using recombinant human CEA biotinylated (RayBiotech, Corners, GA) immobilized on sensor chip SA (Cytiva) at a concentration of 5 µg/ml. M5A and M5A∆C_H_2 mAb constructs were titrated at 8 concentrations (1000, 500, 250, 125, 62.5, 31.25, 15.62, and 7.8 nM). Each run had 300 s contact time with the analyte, 900 s dissociation time with a flow rate of 30 µl/min followed by 2 regeneration steps of 6 M guanidine hydrochloride with a contact time of 60 s each. The sensograms were analyzed for calculating equilibrium dissociation constant (K_D_) using Biacore evaluation software incorporating the 1:1 binding model.

### Conjugation, Radiolabeling and Immunoreactivity

**Fig. 2:**
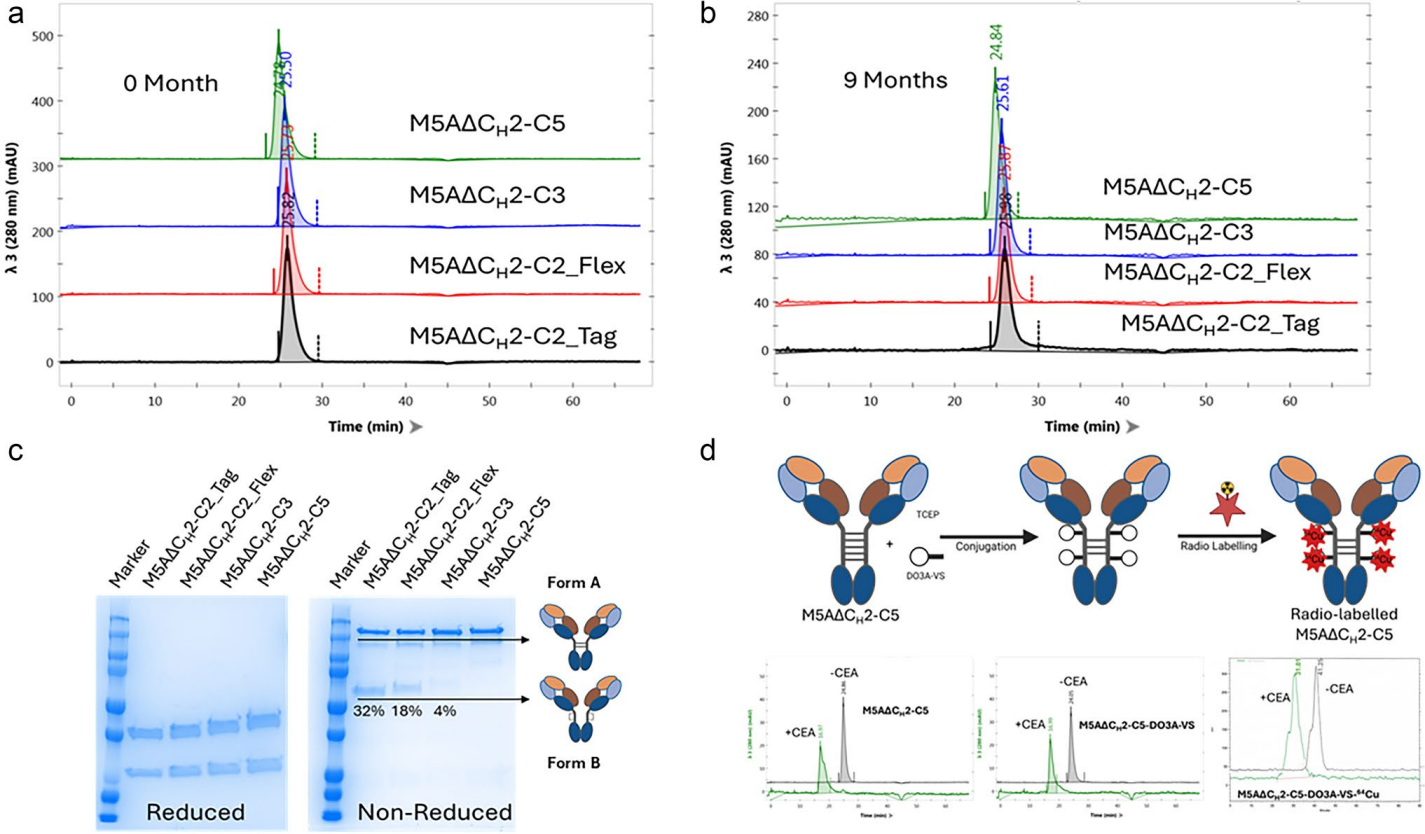
Biochemical and stability analysis of the M5AΔC_H_2 antibody constructs. **a** The purification of the M5AΔC_H_2 antibody constructs resulted in a single peak of the expected molecular size by HPLC-SEC analysis and (**b**) were stable up to 9 months. **c** Analysis of purified M5AΔC_H_2 antibodies on SDS-PAGE under reducing (left panel) and non-reducing conditions (right panel). The M5AΔC_H_2 series showed varying expression of two different isoform forms on the non-reduced gel (Form A and Form B). **d** The M5AΔC_H_2-C5 was mildly reduced, conjugated with DO3A-VS metal chelator, labeled with [^64^Cu] and shown to be immunoreactivity to soluble human CEA by a shift in molecular mass on HPLC-SEC.

The M5A∆CH2-C5 antibody was conjugated with 1,4,7,10 tetraazacyclododecane-1,4,7-triacetic acid trisodium salt-vinyl sulfone (DO3A-VS) as previously described [17]. Briefly, 2 mg of M5A∆CH2-C5 was added to 376 µl of PBS and 24 µl of 10 mM TCEP in a microcentrifuge tube under argon, and incubated at room temperature with rocking for 2 h. The reduced antibody was reacted with 13 µl of DO3A-VS (10 mg/ml stock) and incubated at room temperature with rocking for 2 h. Unconjugated DO3A-VS and TCEP were removed by diafiltration with 0.25 M ammonium acetate, pH 7 (25 DV) using 10 kDa MWCO ultrafiltration membrane in an Amicon stirred cell (Millipore, MA). The DO3A-VS-M5A∆C_H_2-C5 was radiolabeled with [^64^CuCl] (3D Imaging, Little Rock, AR, specific activity 14.1 µCi/µg, in 1 M HEPES for 1 h at 43˚C) The radiolabeling efficiency was 98% by instant thin-layer chromatography. The [^64^Cu]Cu-DO3A-VS-M5A∆C_H_2-C5 was purified by HPLC-SEC. Incubation with soluble CEA (20 molar excess) showed > 95% immunoreactivity by an in vitro molecular weight shift assay and stability study showed the product was stable at least to 72 h by HPLC-SEC as shown in Fig. 2d [17].

### Animal Model and Study Design

All applicable institutional and/or national guidelines for the care and use of animals were followed. All mice were handled in the City of Hope (COH) animal care facility in compliance with COH Institutional Animal Care and Use Committee guidelines, in accordance with the National Institute of Health Office of Laboratory Animal Welfare guidelines. Two animal models were employed bearing subcutaneous colorectal cancer tumors. Six 6-week old female athymic mice were injected with human colorectal cancer LS174T tumors (10^6^ cells in 100 µl per mouse) and six 6-week old female immunocompetent transgenic-CEA (Tg-CEA) mice were injected with MC-38 tumors transfected with the human CEA gene in the flank as previously described [6, 18]. After 12 days, four mice from each group were selected based on the tumor size and injected via the tail vein with 100 µCi/10 µg of [^64^Cu]Cu-D03A-VS-M5A∆C_H_2-C5.

### Imaging and Biodistribution

Serial imaging studies were conducted using β-cube and X-cube (MoleCubes, Ghent, Belgium) for PET and computed tomography (CT) scans, respectively. Mice were kept sedate under isoflurane anesthesia during each imaging session. In both animal model groups, two of the four mice were designated for serial immunoPET imaging at 0, 4, 24, and 48 h post-injection. PET and CT scan images were co-registered using manufacturer-provided software.

Blood clearance was measured by microcapillary sampling of 5 µl of blood from the tail vein at 0, 2, 4, 24, and 48 h post-injection and radioactivity was counted using a calibrated PerkinElmer gamma counter. After the last blood sample or image was acquired at 48 h, all animals were euthanized, necropsy performed, organs weighed (tumor, blood, heart, lung, liver, stomach, small and large intestine, spleen, kidneys, right quadricep muscle, and carcass) and counted for radioactivity. All data are reported as mean values and have been corrected for radioactive decay back to the time of injection, allowing organ uptake to be reported as percent of the injected dose per gram (% IDg^–1^) with standard errors. All statistical analyses were conducted using Prism version 9 (GraphPad Software, San Diego, CA). A two-phase decay nonlinear curve fit with a constrain (plateau = 0) was used to calculate the half-life.

## Results

### Design, Expression, Purification and Characterization of M5A∆C_H_2 Constructs

A series of recombinant anti-CEA humanized M5A antibody fragments were designed for rapid, high-level targeting of gastrointestinal cancers by immunoPET imaging based on the C_H_2 domain-deleted format (named “delta C_H_2”) previously described [3, 5, 7, 23]. For site-specific conjugation of multiple payloads and ensure molecular stability, we engineered in additional disulfide bridges into the IgG hinge domain, adopted from the design of Glaser et al. [10]. Based on human IgG1 and IgG3 hinge sequences, four different variants were designed with two cysteine (C2), three (C3) and five (C5) disulfide bridges along with the C2 construct having two different linkers, Flex and TAG to span the lower hinge to the C_H_3 domain: M5A∆C_H_2-C2_Tag, M5A∆C_H_2-C2_Flex, M5A∆C_H_2-C3_Tag and M5A∆C_H_2-C5_Flex as shown in Fig. 1. Transient mammalian expression of the four M5A∆C_H_2 constructs showed high-level expression (ranging from 65–90 µg/ml). Purification employed a new affinity capture resin, CaptureSelect™ FcXP, which binds to the human C_H_3 antibody domain, resulting in the rapid, high-level purification of the M5A∆C_H_2 series and removal of aggregates [4, 8, 26]. The purified M5A∆C_H_2 antibodies were analyzed for the presence of aggregates by HPLC-SEC, and the results showed that M5A∆C_H_2-C2-Tag, M5A∆C_H_2-C2-Flex, M5A∆C_H_2-C3, and M5A∆C_H_2-C5 contained 10%, 8%, 25%, and 10% aggregates respectively (Supplementary Figure, SF 1). A ceramic hydroxyapatite chromatography (CHT) “polishing” step was incorporated, and HPLC-SEC analysis showed that the two-step purification scheme yielded 100% M5A∆C_H_2 monomer with the expected molecular mass, and the monomers were stable for 1 year (Fig. 2a and 2b).

The purified M5A∆C_H_2 constructs were analyzed by SDS-PAGE gel analysis to determine purity and antibody assembly into covalent disulfide dimers (Fig. 2c). Under reducing condition, all four constructs migrated as two bands, corresponding to the expected light chain and heavy chain molecular weights. However, under non-reducing conditions, heterodimer isoforms were observed, indicating interchain disulfide-bonded isoform A and noncovalently assembled isoform B, as previously described [10, 15]. The M5A∆C_H_2-C2-Tag, M5A∆C_H_2-C2-Flex, M5A∆C_H_2-C3, and M5A∆C_H_2-C5 have 68%, 82%, 96%, and 100% of the disulfide linked isoform A, respectively. M5A∆C_H_2-C5 was determined to be the best candidate in terms of hinge stability, based on the presence of 100% isoform A, and was selected for in vivo PET imaging. The M5A∆C_H_2 constructs were reduced with varying TCEP:protein molar ratio to test the stability. All the samples were completely reduced with a TCEP:protein molar ratio of 15:1 and above. Partial reduction was seen when the samples were reduced using molar ratio of 7.5:1 and lower (Supplementary Figure, SF 2). All M5A∆C_H_2 constructs bound to immobilized CEA with similar affinities as the parent M5A mAb as analyzed by surface plasmon resonance (SPR) on a Biacore X100 instrument (Table 1).

**Table 1:** Kinetic affinity analysis by SPR was performed on the M5A mAb and M5A∆C_H_2 antibody binding to CEA using Langmuir 1:1 binding model. K_d,_ apparent dissociation constant; K_a_ is the association constant; and K_D_ is equilibrium dissociation constant

Sample	K_d_ s^−1^	K_a_ M^−1^ s^−1^	K_D_ pM
M5A mAb	2.1 × 10^–5^	3.4 × 10^–5^	61.7
M5A∆CH2-C5	3.31 × 10^–5^	2.97 × 10^–5^	111.4
M5A∆CH2-C3	2.7 × 10^–5^	3.5 × 10^–5^	77.1
M5A∆CH2-C2-Flex	3.1 × 10^–5^	3.8 × 10^–5^	81.5
M5A∆CH2-C2-Tag	3.2 × 10^–5^	3.8 × 10^–5^	84.2

### Radiolabeling and Immunoreactivity of [^64^Cu]Cu-DO3A-VS-M5A∆C_H_2-C5

For animal PET imaging studies, the M5A∆CH2-C5 was conjugated in the hinge with the thiol-reactive metal chelate, DO3A-VS and radiolabeled with the [^64^Cu] as previously described [17]. The [^64^Cu]Cu-DO3A-VS-M5A∆C_H_2-C5 was purified by SEC-HPLC and shown to be immunoreactive to soluble CEA in preparation for animal studies (Fig. 2d).

### Tumor Targeting, Biodistribution and Pharmacokinetics

**Fig. 3:**
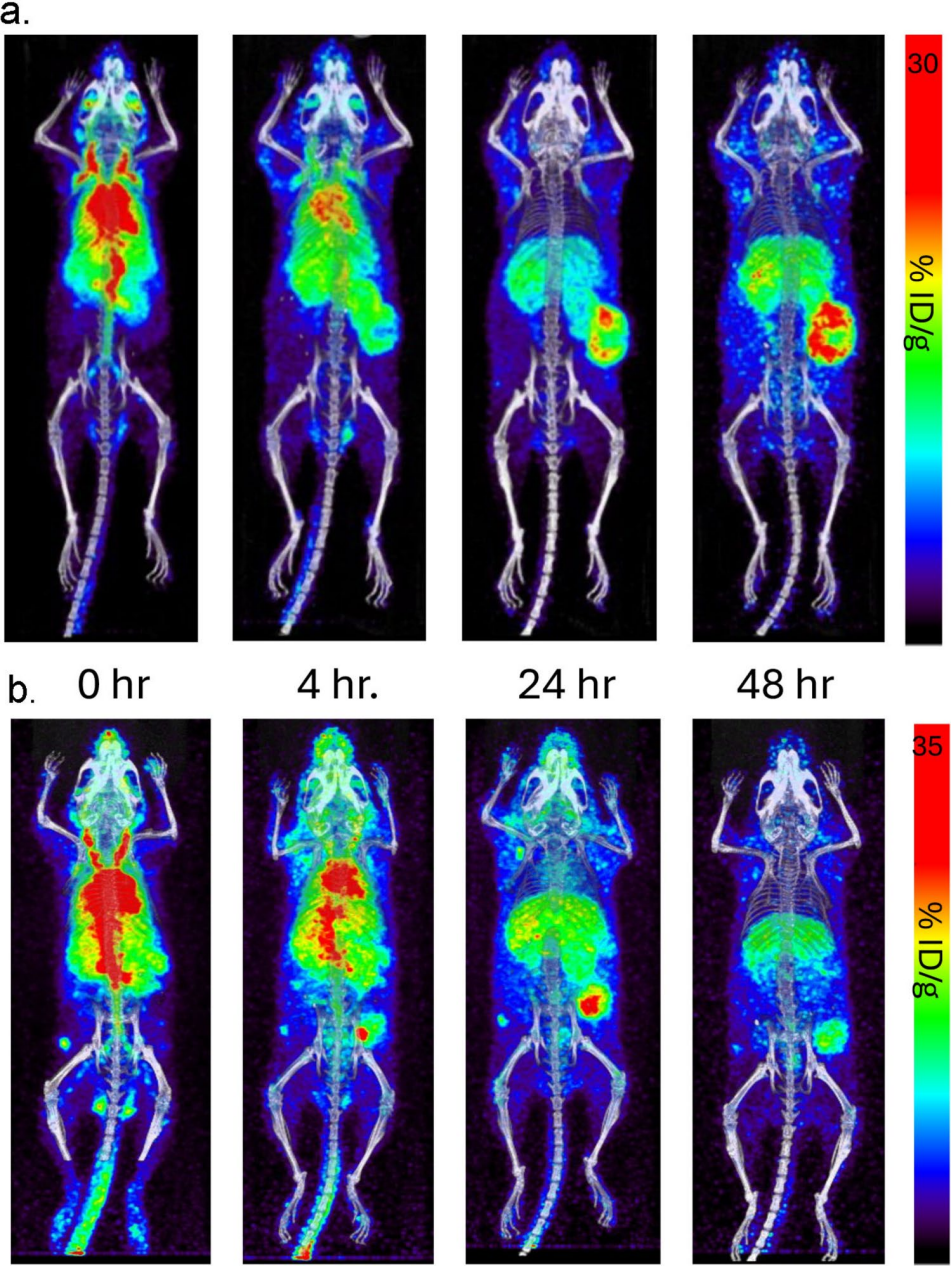
[^64^Cu]Cu-DO3A-VS M5A∆C_H_2-C5 serial PET imaging in colorectal cancer mouse models. [^64^Cu]Cu-DO3A-VS M5A∆C_H_2-C5 antibody PET imaging and terminal biodistribution studies in 2 colorectal cancer mouse models. **a** Serial PET imaging of [^64^Cu]Cu-DO3A-VS M5A∆C_H_2-C5 in athymic mice bearing subcutaneous human colorectal cancer LS-174 T tumors. **b** Serial PET imaging of [^64^Cu]Cu-DO3A-VS M5A∆C_H_2-C5 in immunocompetent CEA-transgenic mice bearing MC-38-CEA tumors.

**Fig. 4:**
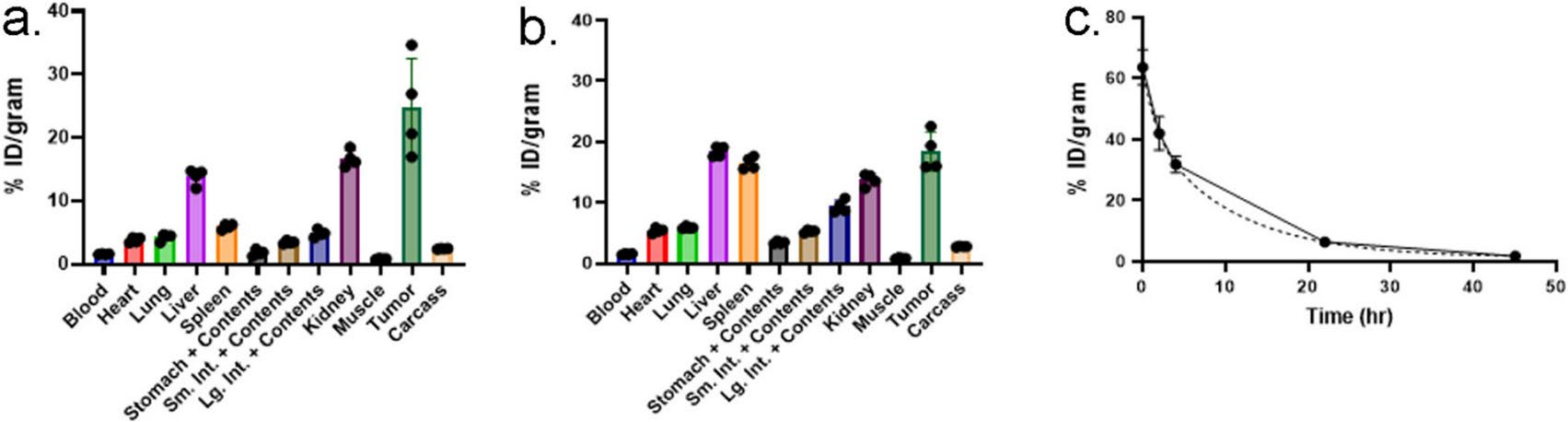
[64Cu]Cu-DO3A-VS M5A∆C_H_2-C5 terminal biodistribution and blood clearance curve. (**a**) Biodistribution of [^64^Cu]Cu-DO3A-VS M5A∆C_H_2-C5 in athymic mouse-LS174T model at 48 h (*n* = 4) (**b**) Biodistribution of [^64^Cu]Cu-DO3A-VS M5A∆C_H_2-C5 in TgCEA-MC38-CEA + mouse model at 48 h (*n* = 4) (**c**) Blood clearance curves of [^64^Cu]Cu-DO3A-VS M5A∆C_H_2-C5 in athymic mice (*n* = 4). The dashed line represents two-phase decay fitting curve.

The [^64^Cu]Cu-DO3A-VS-M5A∆C_H_2-C5 was evaluated for its ability to target CEA-positive tumors in two colorectal cancer mouse models: 1) athymic mice bearing human colorectal cancer LS174T xenografts and 2) immunocompetent CEA-transgenic mice bearing murine colorectal cancer MC-38 cells transfected with human CEA (CEA is only expressed in higher primates, requiring CEA gene transfection) [6, 14]. In this study, four mice from both animal model groups were administered the [^64^Cu]Cu-DO3A-VS-M5A∆C_H_2-C5 by tail vein injection. Two mice from each group were selected for PET serial imaging at 0, 4, 24 and 48 h. [^64^Cu]Cu-DO3A-VS-M5A∆C_H_2-C5 exhibited excellent tumor targeting in both models, as shown in Fig. 3a and 3b. Initially, most of the activity was observed around the thoracic area due to blood pool activity. However, within 3 h, the blood activity decreased, and tumor uptake was visible. Terminal biodistribution was performed immediately after the last PET/CT scan at 48 h. In the LS174T model, the tumor tissue reached 34.6 percent injected dose per gram (% ID/g), the highest of all tissues, followed by kidney (16.6% ID/g) and liver (13.8% ID/g) as shown in Fig. 4a. Similarly, in the Tg-CEA MC-38-CEA + model, the tumor showed the highest accumulation, reaching 22.6% ID/g, followed by liver (18.4% ID/g), spleen (16.5% ID/g), and kidney (13.7% ID/g) as shown in Fig. 4b. Blood samples from LS174T mice (*n* = 4) were collected at 0, 2, 4, 24 and 48 h and radioactivity was counted to determine blood clearance rates. The pharmacokinetics profile of [^64^Cu]Cu-DO3A-VS-M5A∆C_H_2-C5 showed a two-phase clearance with an average 2nd phase half-life (T_1/2β_) of 8.62 h (Fig. 4c).

## Discussion

A series of humanized anti-CEA M5A∆C_H_2 recombinant antibody fragments were developed to explore the potential of additional hinge disulfides to increase thiol-payloads as well as enhanced stability. These M5A∆C_H_2 antibody fragments provided high-level expression in mammalian cell culture, were easily purified using a next generation affinity capture resin and retained the high affinity of the full-length anti-CEA M5A mAb. Biochemical analysis revealed the presence of varying levels of non-covalent assembled species (isoform B), while the M5A∆C_H_2-C5 (five hinge disulfide bridges) demonstrated 100% covalent disulfide-linked isoform A. The M5AΔCH2-C3 exhibited 4% non-covalent isoform-B, while M5AΔCH2-C2-Flex and M5AΔCH2-C2-TAG showed higher levels (18% and 32%, respectively). The M5AΔCH2-C2 variants indicated that the linker composition can have an effect (Flex 18% vs TAG 32%). The hinge in M5AΔCH2-C3 contains an additional cysteine which enhance the yield of isoform-A, while the M5AΔCH2-C5_Flex containing five cysteines eliminated isoform-B. These differences highlight the importance of hinge composition for protein folding, a critical consideration in antibody design for functionality. All M5AΔCH2 series showed comparable stability upon reduction with TCEP (Supplementary Figure, SF 2). The M5A∆C_H_2-C5 was selected for animal studies, conjugated with the reactive thiol-chelate, vinyl sulfone-DO3A, and labeled with [^64^Cu]CuCl_2_. The [^64^Cu]Cu-DO3A-VS-M5A∆C_H_2-C5 demonstrated excellent PET imaging, with high tumor uptake visible at 24 h and rapid blood clearance in two colorectal cancer mouse models. Higher tumor uptake was observed in the human colorectal cancer LS174 tumors compared to MC-38 CEA transfected tumors, presumably due to the difference in antigen density between the tumor models.

Antibody scFv fragments are prone to forming dimers and higher molecular weight species due to Fv cross-pairing, which could impair their clinical efficacy [9, 16]. However, a polishing chromatography step, allowed the isolation of pure monomeric antibody fragments, as confirmed by HPLC-SEC. The study by Glaser et al. also demonstrated similar results with anti-TAG-72 huCC49ΔCH2 fragments, further supporting that optimized hinge sequences, like that in M5AΔCH2-C5, lead to more favorable outcomes [10]. Additionally, more cysteines in the hinge provides additional sites for payload conjugation. Cysteine-based conjugations yield a potent and homogeneous product compared to random-labeled amine chemistry conjugations [32].

Radiolabeled full-length antibodies are often associated with extended blood residence times, which can result in higher marrow irradiation, hampering their therapeutic potential. In contrast, engineered anti-CEA antibody fragments based on T84.66, such as scFvs, diabodies, and minibodies, have been developed to address these limitations by providing faster clearance and higher tumor to background ratios, as observed in multiple preclinical studies [20, 24, 31, 34]. In this study, we explored the delta C_H_2 format with a Fab head group rather than the scFv format employed in the minibody, to avoid the scFv cross pairing observed during production yet retaining the same tumor uptake and blood clearance properties. The [^64^Cu]Cu-DO3A-VS-M5A∆C_H_2-C5 had a blood T_1/2β_ half-life of 8.62 h, falling between the full-length IgG1 M5A mAb and the cT84.66 minibody. Based on the terminal biodistribution analysis, the [^64^Cu]Cu-DO3A-VS-M5A∆C_H_2-C5 cleared primarily via the liver and kidney in the athymic mice; however, in the immunocompetent Tg-CEA mice, activity can also be found in the spleen.

The M5A∆C_H_2-C5 antibody provides a promising approach to balancing clearance rates with high tumor localization, offering potential benefits not only for diagnostic imaging but also for future therapeutic applications.

## Conclusion

In this study, we successfully designed and produced a series of recombinant humanized anti-CEA M5A∆CH2 antibody fragments for immunoPET imaging of colorectal cancer. The engineered M5A∆CH2 constructs, incorporating additional disulfide bridges in the hinge domain, provided multiple sites for site-specific conjugation and demonstrated enhanced stability. The [^64^Cu]Cu-DO3A-VS-M5A∆C_H_2-C5 PET imaging demonstrated high tumor targeting and favorable pharmacokinetics in two CEA-positive colorectal cancer mouse models. In summary, the humanized recombinant anti-CEA M5A∆CH2-C5 antibody has the potential to be a valuable diagnostic tool for immunoPET imaging of colorectal cancer in the clinic.

## Supplementary Information

Below is the link to the electronic supplementary material.

Supplementary file1 HPLC-SEC Superdex 200 analysis after FcXP purification of M5AΔC_H_2 antibody fragments, percentage value next to the peak on chromatogram representing the percent of monomer present in the sample. (JPG 122 KB)

ESM 2 (PNG 415 KB)

High Resolution Image The M5A∆C_H_2 constructs were subjected to reduction by TCEP at varying TCEP: protein ratios. All the constructs were reduced completely at a TCEP: protein ratio of 15:1 or higher (left gel). At lower ratios partially reduced antibody can be seen with various intermediate fragments (right gel). (TIF 767 KB)

## Data Availability

The data that support the findings of this study are openly available upon request to the corresponding author.
