# Supplementary figures and images for: Development of Anti-CEA CH2 Domain-Deleted Antibody (M5A∆CH2) for the PET Imaging of Colorectal Cancer
Source: Mol Imaging Biol. 2025 Mar 14;27(2):192–200. doi: 10.1007/s11307-025-01997-3 (PMC12062029; doi:10.1007/s11307-025-01997-3)

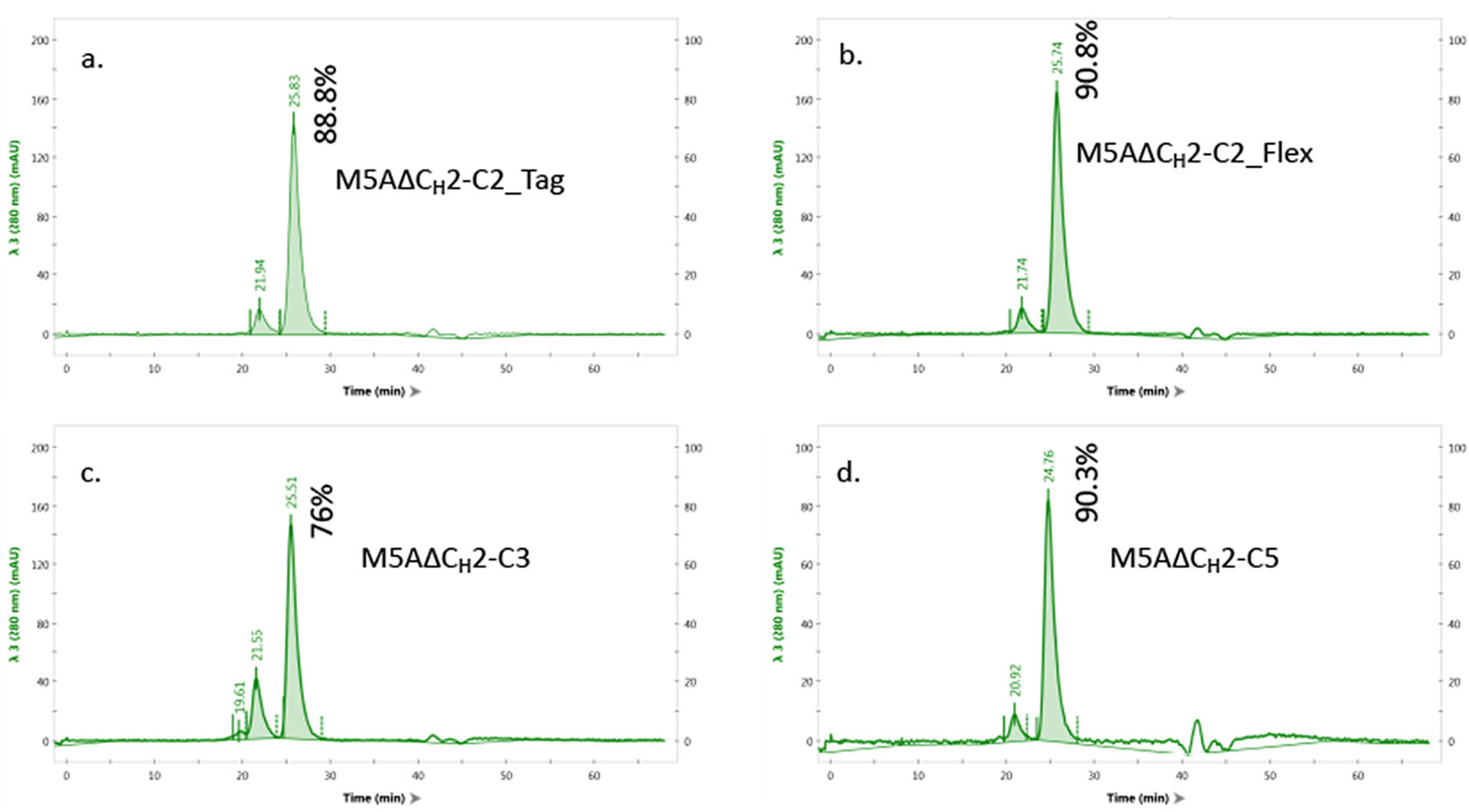

Supplement: Supplementary file 1 — Supplementary file1 HPLC-SEC Superdex 200 analysis after FcXP purification of M5AΔCH2 antibody fragments, percentage value next to the peak on chromatogram representing the percent of monomer present in the sample. (JPG 122 KB) [file 11307_2025_1997_MOESM1_ESM.jpg]

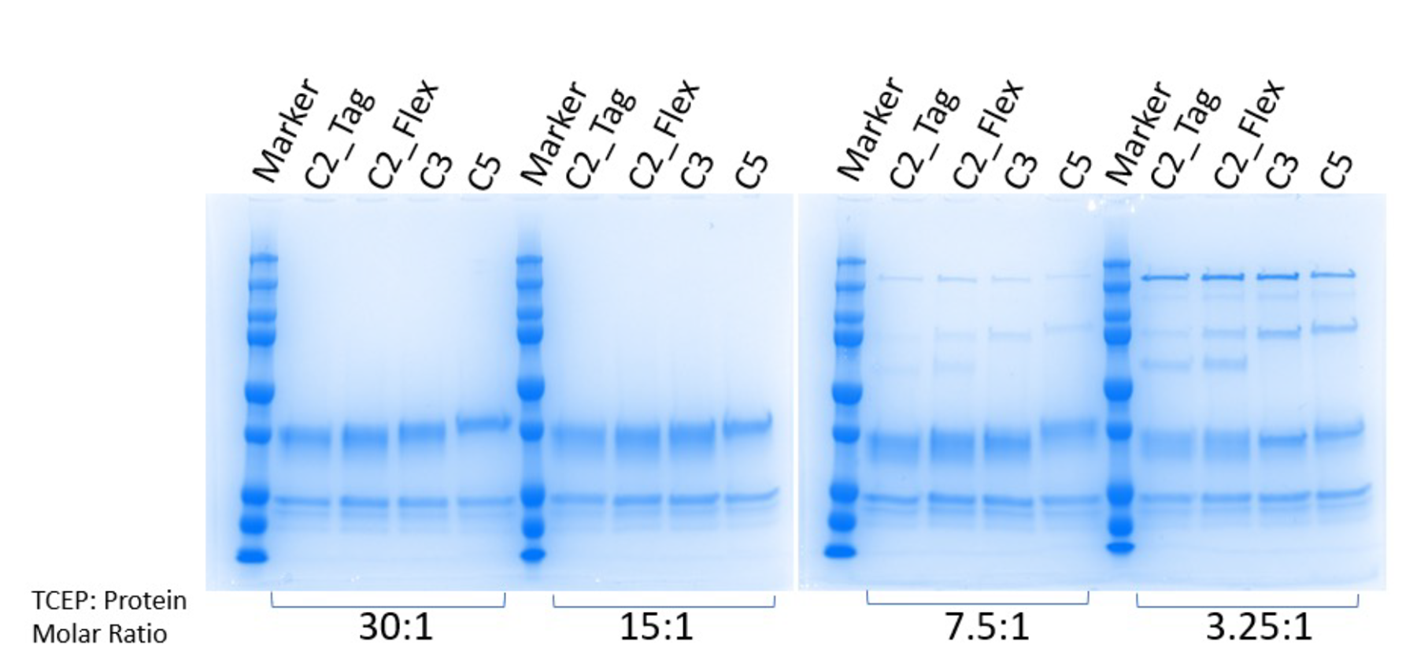

Supplement: Supplementary file 2 — (PNG 415 KB) [file 11307_2025_1997_Fig5_ESM.png]

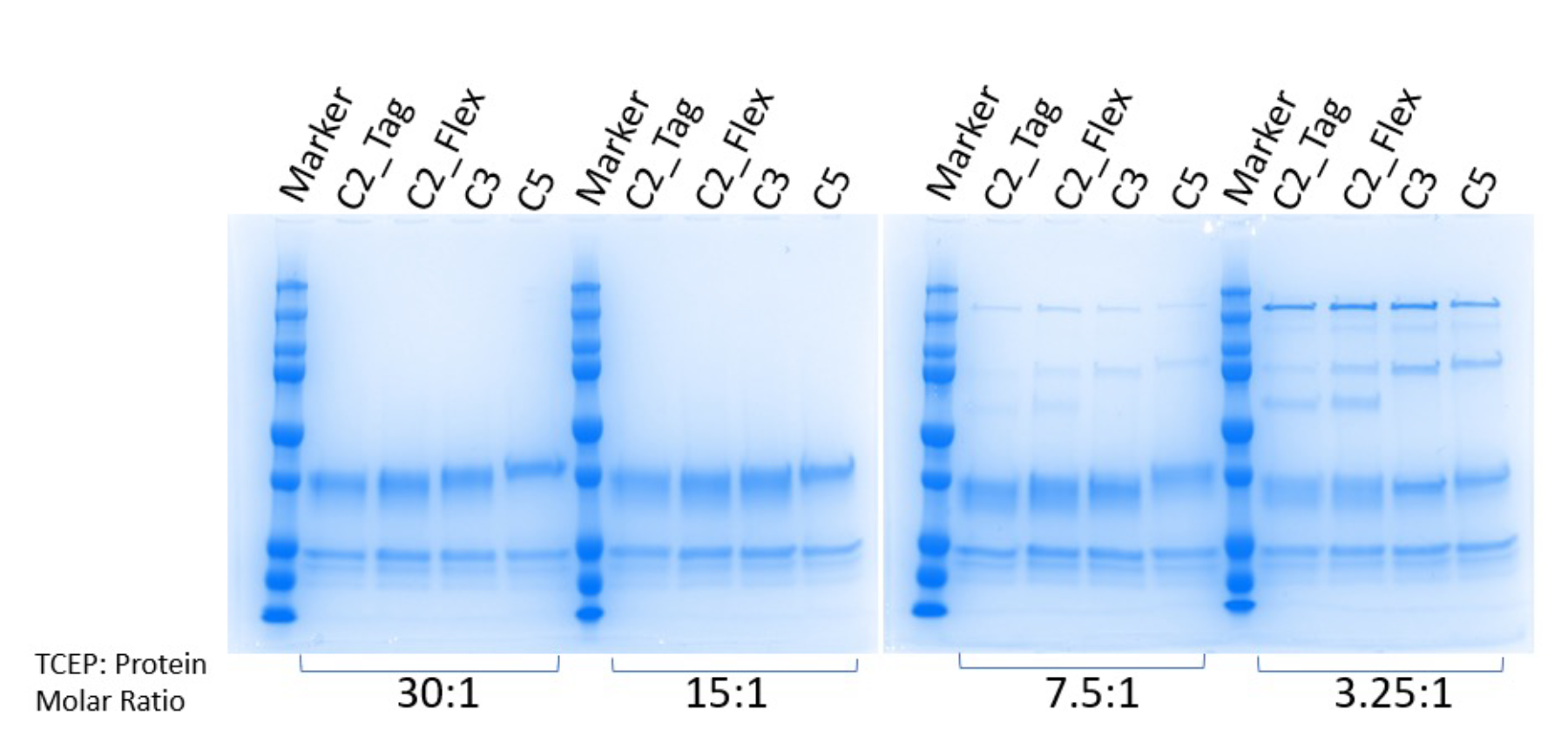

Supplement: Supplementary file 3 — High Resolution Image The M5A∆CH2 constructs were subjected to reduction by TCEP at varying TCEP: protein ratios. All the constructs were reduced completely at a TCEP: protein ratio of 15:1 or higher (left gel). At lower ratios partially reduced antibody can be seen with various intermediate fragments (right gel). (TIF 767 KB) [file 11307_2025_1997_MOESM2_ESM.tif]
